# Safety and aesthetic outcomes of double purse-string suture nipple reconstruction in early breast cancer patients undergoing nipple resection and endoscopic skin-sparing mastectomy with breast reconstruction

**DOI:** 10.3389/fonc.2024.1462850

**Published:** 2024-09-30

**Authors:** Hui Dai, Kawun Chung, Faqing Liang, Yanyan Xie, Qing Zhang, Mengxue Qiu, Huanzuo Yang, Jiao Zhou, Yu Feng, Zhenggui Du

**Affiliations:** ^1^ Department of General Surgery, West China Hospital, Sichuan University, Chengdu, China; ^2^ Breast Center, West China Hospital, Sichuan University, Chengdu, China; ^3^ Department of Thyroid and Breast Surgery, The First People’s Hospital of Ziyang, Sichuan University, Ziyang, China; ^4^ Department of General Surgery, The Fourth People’s Hospital of Sichuan Province, Chengdu, China

**Keywords:** double purse-string suture, nipple reconstruction, endoscopic skin-sparing mastectomy, breast reconstruction, breast cancer

## Abstract

**Background:**

The current surgical methods for managing incisions after nipple excision in breast reconstruction patients are limited. However, double purse-string suture (DPS) shows promise in the treatment of nipple excision. This study aimed to investigate the safety and aesthetic outcomes of DPS nipple reconstruction in early breast cancer patients who underwent endoscopic skin-sparing mastectomy (E-SSM) and breast reconstruction.

**Methods:**

We retrospectively analyzed the clinical data of 87 early breast cancer patients with nipple excision who underwent E-SSM with breast reconstruction. According to the suture methods of nipple incision, all patients were divided into the spindle suture (SS) group, single purse-string suture (SPS) group, and DPS group, with SS and SPS groups combined as the traditional suture (TS) group. Then, we compared the groups’ differences in aesthetic outcomes, surgical safety, and oncological safety.

**Results:**

A total of 87 patients with 88 breasts were enrolled in this study (SS n=17, SPS n=21, DPS n=50). Patients in the DPS group had significantly better nipple reconstruction satisfaction, Harris scale and any complications incidence than the TS group (all p <0.05). For nipple reconstruction satisfaction and any complication, the adjusted OR (95%CI) of the DPS group were 6.314(1.095-36.415) (p=0.039) and 0.124(0.018-0.863) (p=0.035) compared with the SS group. One patient in the SS group had vertebral metastases, and no recurrence, metastasis, or death has been observed in the other two groups during the follow-up period.

**Conclusions:**

DPS is an effective and safe nipple reconstruction procedure for patients undergoing E-SSM with breast reconstruction, delivering excellent aesthetic outcomes.

## 1 Introduction

Endoscopic breast reconstruction is being performed more and more frequently in patients with early breast cancer for its better aesthetic results and lower postoperative complication rates (1–13). The nipple-areola complex (NAC) is an essential anatomical part of the woman, and the excision of NAC has considerable aesthetic and psychological consequences (14, 15). Whenever possible, the surgeon spares the NAC during mastectomy (10, 16–18). However, when there are situations such as a direct extension of the tumor to the nipple on imaging or pathology, the resection of the nipple or even the NAC is almost unavoidable. Even when there are microcalcifications close to the subareolar region and a positive nipple discharge, the NAC may also be removed (16, 19). Then, surgeons must address two critical concerns, the safe closure and favorable aesthetic effect.

Currently, two traditional suture methods are used to close the nipple-areola excision incision: the spindle suture (SS) and the single purse-string suture (SPS). However, they both have some disadvantages. For example, neither SS nor SPS can only close the incision. The higher incision tension in SS and the insufficient strength of incision closure in SPS are likely to lead to complications such as poor incision healing or even incision disruption. Besides, the breast shape is often deformed after SS. Therefore, it is of great clinical importance to explore a safe and aesthetically pleasing method of nipple incision management.

After consideration and practice, our team found the double purse-string suture (DPS) to be a superior method. It consists of rows of inner and outer double purse-string sutures around the nipple incision, which can not only close the incision tightly with less tension but also have the effect of nipple reconstruction utilizing the residual areola around the incision. Previous studies have also found that purse-string sutures can improve the projection of the nipple and the areola area (20–22). However, few studies have explored the use of DPS in nipple-resected patients with breast reconstruction. Therefore, we conducted this retrospective study to investigate the safety and aesthetic outcomes of DPS in early breast cancer patients who underwent nipple resection and E-SSM with breast reconstruction.

## 2 Patients and methods

### 2.1 Study design and patients

Eighty-seven patients were included in this study, undergoing nipple resection, E-SSM with breast reconstruction from November 2015 to April 2024 at West China Hospital, Sichuan University, China. The inclusion criteria: i) 18 - 70 years old; ii) early invasive breast cancer in stage I or II or ductal carcinoma *in situ* or prophylactic mastectomy; iii) the tumor did not invade the skin and chest wall confirmed by physical examination and breast MRI; iv) the maximum preoperative diameter of invasive cancer was ≤5 cm. The exclusion criteria: i) disorder of the coagulation mechanism or tendency to bleed; ii) severe cardiopulmonary dysfunction; iii) inability to tolerate general anesthesia; iv) immunocompromised; v) breast cancer in pregnancy (3, 23). No male patients met the included criteria so all the participants were female. The clinical data of the 87 patients were collected from the prospectively maintained database, follow-up records, medical records, and phone call reviews. According to the suture methods of nipple incision, all patients were divided into three groups: SS group, SPS group, and DPS group, with SS and SPS groups combined as the traditional suture (TS) group. This study was approved by the Biomedical Ethics Committee of West China Hospital, Sichuan University (No. 2021-863). Patients agreed to and signed consent to publish their photographs or videos.

### 2.2 Surgical procedures

**Figure 1:**
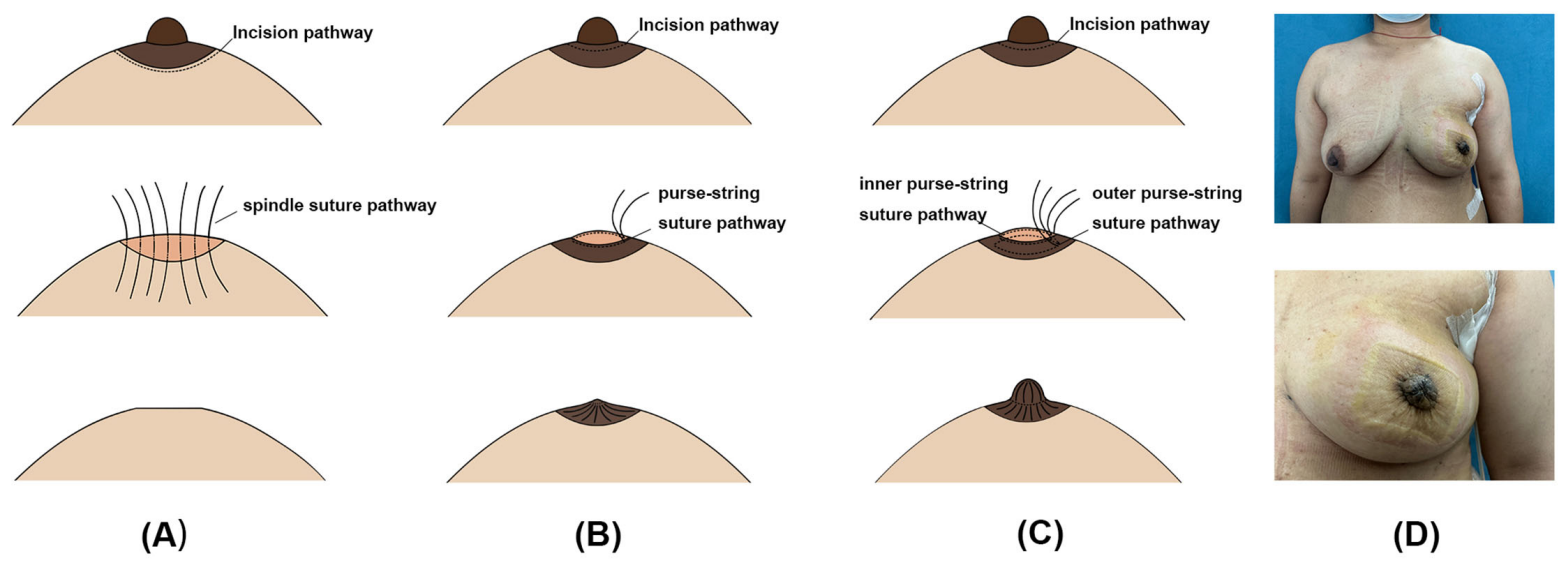
Surgical procedures of spindle suture (SS), single purse-string suture (SPS) and double purse-string suture (DPS). **(A–C)** The demonstration of surgical procedures for SS, SPS and DPS. **(D)** The 2 weeks postoperative breast images of a patient with DPS.

Nipple excision was performed when intraoperative pathology shows direct tumor extension to the nipple or there is a preoperative history of bloody nipple spillage (16, 19). The three suturing methods of nipple incision closure are respectively shown in **Figure 1A**. SS is performed through a fusiform incision closed by subcutaneous intermittent sutures and the followed intracutaneous continuous sutures. SPS procedure involves making a circular incision and closing it with a single purse-string suture. The DPS procedure also involves making a circular incision, which is closed by inner and outer purse-string sutures about 1cm apart. The outer purse tightens the nipple incision, reduces tension and creates a columnar nipple structure, while the inner purse is used to close the incision with low tension. An image of a patient two weeks after DPS is shown in **Figure 1**. The specific steps involved in the DPS procedure are clearly demonstrated in **Video 1**. The surgical procedures schematics and image layouts were completed using Adobe Illustrator 2024 and Adobe Photoshop CC 2019.

### 2.3 Postoperative management

After surgery, the patient must wear a breast contouring garment for 3 months. In the DPS group, petal-like holes will be cut in the nipple area of the breast contouring garment to decrease pressure on the reconstructed nipple. The reconstructed nipple will be covered with a layer of Vaseline sterile gauze before bandaging to promote wet healing. (24–26) The drain from the mastectomy area was removed when there was less than 30 mL of discharge for 3 consecutive days and no bleeding (2, 27, 28).

### 2.4 Follow-up

Patients were followed up in the outpatient clinic at 2 weeks, 1 month, 3 months, and every 6 months after discharge until May 1, 2024. Follow-up time was calculated from the end of the patient’s operation to the last follow-up visit. We recorded the postoperative examination results and complications, evaluated the surgical effect, survival status, recurrence and metastasis of the patients, and guided the patients to evaluate the satisfaction of the reconstructed nipple and fill in the Harris scale, Scar-Q questionnaire. We recorded the latest follow-up data of the above programs. Preoperative and postoperative treatments, including standard regimens and treatment courses, were conducted per breast cancer treatment guidelines.

Breast ptosis was graded according to the Regnault grading (29). The pathological staging was performed per the eighth edition of the American Joint Committee on Cancer (AJCC) TNM staging system for breast cancer (30). Capsular contracture was evaluated based on Baker grading (31). The Harris scale was used to record patients’ subjective judgment of breast symmetry and evaluate the appearance of the reconstructed breast relative to the contralateral side (32). Scar-Q mainly assesses scar appearance (such as size, color, and contour) from the subjective perspective of patients (33). Postoperative complications include major and minor complications, which are classified with reference to the Clavien–Dindo classification (CDC) (34). Grade III and IV complications are major and require additional surgical intervention. Grade I and II complications are minor and do not require surgical management. Recurrence and metastasis were determined based on imaging tests and pathological biopsies conducted during the patient’s visit.

### 2.5 Statistical analysis

All statistical analyses were performed with IBM SPSS Statistic 27. The Shapiro-Wilk test was used to test the normality of the quantitative data distribution. Quantitative data that were normally distributed or not were expressed as mean ± standard deviation $\left(\bar{\text{X}}\pm \text{s}\right)$ or median (interquartile range) [M (P25, P75)], respectively. Qualitative data were expressed as frequency (percentage) [n (%)]. Differences in patient characteristics, postoperative complications, and aesthetic outcomes between different suture method groups in the whole population or subgroups were compared using Student’s t-test, One-way ANOVA, Mann-Whitney U test, Chi-squared test, or Fisher’s precision probability test. To further explore the relationship between different nipple incision suture methods and aesthetic outcomes and postoperative complications in different age subgroups, we classified the age ≤ 40 and > 40 years subgroups based on the mean of age. Similarly, radiotherapy and non-radiotherapy groups were explored. Univariate binary logistic regression analyses and mixed-effects logistic regression model were used to test the effects of suture methods on the odds ratios (ORs) of nipple reconstruction satisfaction, Harris scale and any complications. The level of significance was taken to be p≤ 0.05 for all statistical tests.

## 3 Results

### 3.1 Patient characteristics

In this study, 88 breasts of 87 patients were involved, with 17, 21, and 50 breasts in the SS, SPS and DPS groups. The mean ages of the SS, SPS, and DPS groups were 40.47 ± 9.59, 41.86 ± 7.16, and 41.28 ± 7.81 years. Compared to the TS group, the DPS group had longer follow-up time, a higher BMI and preoperative cup size, lower durations of operations and hospitalization, a higher percentage of 24h-admission operation, and a lower extent of nipple resection (all p < 0.05). The median operation duration of the DPS group was 178.50 minutes, whereas it was 225.00 minutes in the TS group. The percentage of 24h-admission operation was 38.0% in the DPS group compared to 10.5% in the TS group. 86.0% and 50.0% of patients in the DPS and TS groups removed the nipple and partial areola. The characteristics of the three groups are summarized in **Table 1**.

**Table 1:** Characteristics of patients with different nipple incision suture methods.

Variables	TS (n=38)		DPS (n=50)	p1	p2
	SS (n=17)	SPS (n=21)			
Follow-up time(months), median (IQR)	33.27(28.67-35.60)	37.53(30.73-48.47)	30.35(20.33-38.63)	0.040	0.031
Age(year), mean ± SD	40.47 ± 9.59	41.86 ± 7.16	41.28 ± 7.81	0.869	0.980
BMI (kg/m²), median (IQR)	20.96(19.92-22.55)	20.96(19.72-22.43)	22.31(20.57-24.97)	0.057	0.019
Hypertension, n (%)				0.821	0.384
Yes	0(0.0)	1(4.8)	4(8.0)		
No	17(100.0)	20(95.2)	46(92.0)		
Breast ptosis, n (%)				0.831	0.466
None	10(58.8)	14(66.7)	27(54.0)		
Pseudo	2(11.8)	1(4.8)	5(10.0)		
I	4(23.5)	6(28.6)	11(22.0)		
II	1(5.9)	0(0.0)	6(12.0)		
III	0(0.0)	0(0.0)	1(2.0)		
Preoperative cup size, n (%)				0.099	0.048
A	3(17.6)	4(19.0)	3(6.0)		
B	11(64.7)	9(42.9)	20(40.0)		
C	2(11.8)	7(33.3)	17(34.0)		
≥D	1(5.9)	1(4.8)	10(20.0)		
Surgical site, n (%)				0.147	0.903
Unilateral	11(64.7)	19(90.5)	40(80.0)		
Bilateral	6(35.3)	2(9.5)	10(20.0)		
Operation duration(min), median (IQR)	190.50(170.00-279.00)	231.00(172.50-345.50)	178.50(156.00-257.00)	0.072	0.024
Hospitalization duration(day), median (IQR)	7.00(6.00-7.00)	7.00(6.00-8.00)	6.00(1.00-7.00)	0.017	0.006
Operation type, median (IQR)				0.010	0.004
Ward operation	14(82.4)	20(95.2)	31(62.0)		
24h-admission operation	3(17.6)	1(4.8)	19(38.0)		
Axillary surgery, n (%)				0.316	0.582
Sentinel lymph node biopsy	12(70.6)	10(47.6)	26(52.0)		
Lymph node dissection	5(29.4)	11(52.4)	24(48.0)		
Reconstruction methods, n (%)				0.247	0.074
Prepectoral breast reconstruction	3(17.6)	2(9.5)	19(38.0)		
Subpectoral breast reconstruction	7(41.2)	10(47.6)	15(30.0)		
Dual plane breast reconstruction	5(29.4)	7(33.3)	11(22.0)		
Latissi-mus dorsi flap breast reconstruction	2(11.8)	2(9.5)	5(10.0)		
Implant, n (%)				0.968	1.000
Latissi-mus dorsi flap	1(5.9)	0(0.0)	1(2.0)		
Prosthesis	14(82.4)	18(85.7)	42(84.0)		
Expander	1(5.9)	1(4.8)	3(6.0)		
Latissi-mus dorsi flap and prosthesis	1(5.9)	2(9.5)	4(8.0)		
Implant size, median (IQR)	215.00(180.00-260.00)	267.50(200.00-307.50)	270.00(232.50-342.50)	0.122	0.113
Extent of NAC resection				0.001	< 0.001
Nipple and Partial areola	9(52.9)	10(47.6)	43(86.0)		
Nipple and Whole areola	8(47.1)	11(52.4)	7(14.0)		
Postoperative pathology, n (%)				0.822	1.000
Others	2(11.8)	1(4.8)	3(6.0)		
Carcinoma in situ	2(11.8)	1(4.8)	5(10.0)		
Invasive carcinoma	13(76.5)	19(90.5)	42(84.0)		
pT stage (NA=9), n (%)				0.455	0.549
Tis and T1	8(57.1)	10(55.6)	31(66.0)		
T2	6(42.9)	6(33.3)	15(31.9)		
T3	0(0.0)	2(11.1)	1(2.1)		
pN stage (NA=9), n (%)				0.861	0.607
Positive	5(35.7)	7(38.9)	15(31.9)		
Negative	9(64.3)	11(61.1)	32(68.1)		
ER(NA=5), n (%)				0.601	0.354
Positive	12(75.0)	14(70.0)	38(80.9)		
Negative	4(25.0)	6(30.0)	9(19.1)		
PR(NA=7), n (%)				0.766	0.676
Positive	12(80.0)	14(70.0)	36(78.3)		
Negative	3(20.0)	6(30.0)	10(21.7)		
HER-2(NA=20), n (%)				0.539	0.661
Positive	5(38.5)	4(22.2)	9(24.3)		
Negative	8(61.5)	14(77.8)	28(75.7)		
Ki-67(NA=7), n (%)				0.278	0.120
<20%	9(56.3)	10(50.0)	16(35.6)		
≥20%	7(43.8)	10(50.0)	29(64.4)		
Neoadjuvant chemotherapy, n (%)				0.650	0.484
Yes	5(29.4)	5(23.8)	10(20.0)		
No	12(70.6)	16(76.2)	40(80.0)		
Adjuvant chemotherapy, n (%)				0.396	0.807
Yes	6(35.3)	12(57.1)	25(50.0)		
No	11(64.7)	9(42.9)	25(50.0)		
Adjuvant radiotherapy, n (%)				0.854	0.984
Yes	5(29.4)	8(38.1)	17(34.0)		
No	12(70.6)	13(61.9)	33(66.0)		
Adjuvant endocrinotherapy, n (%)				0.639	0.486
Yes	13(76.5)	14(66.7)	32(64.0)		
No	4(23.5)	7(33.3)	18(36.0)		
Anti-HER-2 therapy, n (%)				0.894	0.529
Yes	1(5.9)	2(9.5)	6(12.0)		
No	16(94.1)	19(90.5)	44(88.0)		

TS, traditional suture; SS, spindle suture; SPS, single purse-string suture; DPS, double purse-string suture; BMI, body mass index; ER, estrogen receptor; PR, progesterone receptor; HER-2, human epidermal growth factor receptor-2.

Variables above were compared differences between groups based on breasts, with sample size equal to the number of breasts.

p1: SS vs. SPS vs. DPS; p2: TS vs. DPS.

### 3.2 Aesthetic evaluation

As shown in **Table 2**, the distribution of nipple reconstruction satisfaction in the DPS group was significantly different from that of the TS group (p=0.002). The “very satisfied” percentage were 36.0%, 11.8%, 4.8% in the DPS, SS and SPS groups. For the Harris scale, it reported a 48.0% excellent rate in the DPS group, compared to 9.5% and 35.3% in the SPS and SS groups(p=0.023). The Scar-Q scores were 68.50(46.00-84.00) and 68.00(55.00-80.00) in the DPS and SPS group, which were higher than 47.50(44.00-66.00) in the SS group(p=0.418). The difference in the incidence of implant-related complications among the three groups were insignificant (all p > 0.05). In the age ≤ 40 years subgroup, there was a significant difference in nipple reconstruction satisfaction between TS and DPS groups (p=0.002) (**Supplementary Tables 1**, **2**). In the non-radiotherapy group, the nipple reconstruction satisfaction and Harris score in DPS were significantly different from those in the TS group (all p < 0.05) (**Supplementary Tables 3**, **4**).

**Table 2:** Comparison of aesthetic outcomes and aesthetic complications between patients with different nipple incision suture methods.

Variables	TS (n=38)		DPS(n=50)	p1	p2
	SS (n=17)	SPS (n=21)			
Nipple reconstruction satisfaction, n (%)				0.007	0.002
Very Satisfied	2(11.8)	1(4.8)	18(36.0)		
Relatively Satisfied	4(23.5)	8(38.1)	16(32.0)		
Relatively dissatisfied	6(35.3)	7(33.3)	14(28.0)		
Very dissatisfied	5(29.4)	5(23.8)	2(4.0)		
Harris scale, n (%)				0.023	0.024
Excellent	6(35.3)	2(9.5)	24(48.0)		
Good	4(23.5)	5(23.8)	10(20.0)		
Fair	5(29.4)	11(52.4)	15(30.0)		
Poor	2(11.8)	3(14.3)	1(2.0)		
Scar-Q(NA=11), median (IQR)	47.50(44.00-66.00)	68.00(55.00-80.00)	68.50(46.00-84.00)	0.418	0.357
Implant-related complications, n (%)					
Capsular contracture, n (%)				0.424	0.239
None	9(52.9)	13(61.9)	35(70.0)		
I	0(0.0)	2(9.5)	4(8.0)		
II	6(35.3)	0(0.0)	5(10.0)		
III	2(11.8)	6(28.6)	6(12.0)		
IV	0(0.0)	0(0.0)	0(0.0)		
Rippling, n (%)				0.432	0.432
Yes	0(0.0)	1(4.8)	0(0.0)		
No	17(100.0)	20(95.2)	50(100.0)		
Outliner of the implant wrinkling, n (%)				1.000	1.000
Yes	0(0.0)	1(4.8)	2(4.0)		
No	17(100.0)	20(95.2)	48(96.0)		

TS, traditional suture; SS, spindle suture; SPS, single purse-string suture; DPS, double purse-string suture.

p1: SS vs. SPS vs. DPS; p2: TS vs. DPS.

**Figure 2:**
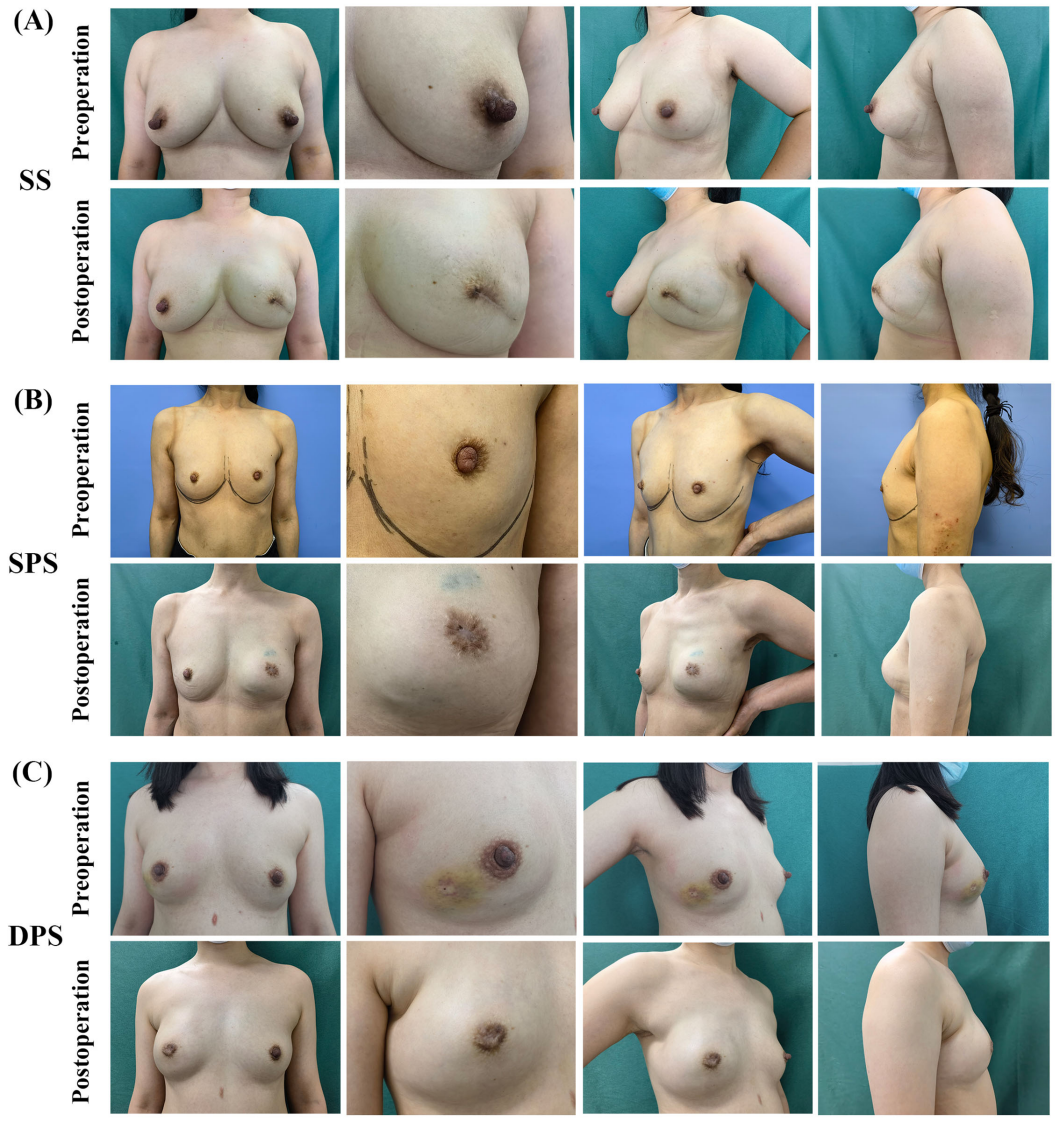
Pre- and post-operative images of patients with SS, SPS, DPS. **(A)** Preoperative and 10-month postoperative images of a patient who underwent spindle suture and endoscopic skin-sparing mastectomy and prepectoral direct-to-implant breast reconstruction. **(B)** Preoperative and 12-month postoperative images of a patient who underwent single purse-string suture and endoscopic skin-sparing mastectomy and direct-to-implant breast reconstruction with Latissimus Dorsi Flap. **(C)** Preoperative and 11-month postoperative images of a patient who underwent double purse-string suture and endoscopic skin-sparing mastectomy and prepectoral direct-to-implant breast reconstruction.

**Table 3** presents ORs and 95%CIs for satisfied with the reconstructive nipple or satisfied Harris scale according to suture methods with age, BMI, breast ptosis, preoperative cup size, operation time, reconstruction method, axillary surgery, extent of nipple resection, implant type, implant size, adjuvant radiotherapy and follow-up time as the adjusted factors. The results revealed that compared with the SS group, the DPS group had significantly higher odds of satisfied nipple reconstruction satisfaction with an adjusted OR of 6.314 (95%CI: 1.095-36.415, p=0.039). However, there was no significant difference between the two groups on the Harris scale, with an adjusted OR of 0.917(DPS vs. SS, 95%CI: 0.167-5.043, p=0.921). Pre- and post-operative images of patients with the three suture methods are shown in **Figure 2**.

**Table 3:** OR and 95%CI for satisfied with the reconstructive nipple or satisfied Harris scale according to nipple incision suture methods.

Variables	Nipple reconstruction satisfaction				Harris scale			
	Rude OR (95%CI)	p	Adjusted OR (95%CI)	p	Rude OR (95%CI)	p	Adjusted OR (95%CI)	p
		0.029		0.061		0.033		0.634
SS (n=17)	Reference		Reference		Reference		Reference	
SPS(n=21)	1.375 (0.368-5.136)	0.636	1.387 (0.212-9.072)	0.733	0.350 (0.093-1.317)	0.120	0.437 (0.059-3.228)	0.417
DPS(n=50)	3.896 (1.223-12.411)	0.021	6.314 (1.095-36.415)	0.039	1.487 (0.479-4.623)	0.493	0.917 (0.167-5.043)	0.921

OR: Odds ratio; 95%CI: 95% confidence interval; DPS, double purse-string suture; SS, spindle suture; TS, traditional suture; BMI, body mass index; ER, estrogen receptor; PR, progesterone receptor; HER-2, human epidermal growth factor receptor-2.

Satisfied with the Reconstructive Nipple: the nipple reconstruction satisfaction is very satisfied or relatively satisfied. Satisfied Harris Scale: the Harris scale is excellent or good.

Adjusted OR (95%CI): adjust for age, BMI, breast ptosis, preoperative cup size, operation time, reconstruction method, axillary surgery, extent of nipple resection, implant type, implant size, adjuvant radiotherapy and follow-up time.

### 3.3 Postoperative complications

The DPS group had a significantly lower incidence of any complications (20.0%) compared to the TS group (42.1%) (p=0.024). The incidence of any complications in the SS and SPS groups was 41.2% and 42.9%. Notably, the incidence of major complications in the SPS group was higher compared to the SS and DPS groups, with a rate of 14.3% (p=0.018). Within the SPS group, there were three cases of major complication events, including two surgical site infections that required surgical management, and one case of implant removal. The difference in incidence of minor complications among the three groups was insignificant (p=0.185). The details of postoperative complications are shown in **Table 4**.

**Table 4:** Comparison of postoperative complications between patients with different nipple incision suture methods.

Variables	TS (n=38)		DPS (n=50)	p1	p2
	SS (n=17)	SPS (n=21)			
Any complications				0.079	0.024
Yes	7(41.2)	9(42.9)	10(20.0)		
No	10(58.8)	12(57.1)	40(80.0)		
Major complications				0.018	0.077
Yes	0(0.0)	3(14.3)	0(0.0)		
No	17(100.0)	18(85.7)	50(100.0)		
Minor complications				0.185	0.079
Yes	7(41.2)	7(33.3)	10(20.0)		
No	10(58.8)	14(66.7)	40(80.0)		
Seroma	5(29.4)	4(19.0)	5(10.0)	0.145	0.082
Incision disruption				0.090	0.184
Dressing change	0(0.0)	1(4.8)	0(0.0)		
Requiring surgical revision	0(0.0)	1(4.8)	0(0.0)		
Skin flap ischemia/necrosis				0.193	0.432
Mild	1(5.9)	0(0.0)	0(0.0)		
Severe	0(0.0)	0(0.0)	0(0.0)		
Surgical site infection				0.377	0.250
Treated with oral antibiotics	3(17.6)	2(9.5)	4(8.0)		
Treated with intravenous antibiotics	0(0.0)	1(4.8)	2(4.0)		
Requiring surgical revision	0(0.0)	2(9.5)	0(0.0)		
Hematoma/hemorrhage				0.432	0.432
Treated with pressure dressing	0(0.0)	1(4.8)	0(0.0)		
Requiring surgical revision	0(0.0)	0(0.0)	0(0.0)		
Implant removal	0(0.0)	1(4.8)	0(0.0)	0.432	0.432

TS, traditional suture; SS, spindle suture; SPS, single purse-string suture; DPS, double purse-string suture.

p1: SS vs. SPS vs. DPS; p2: TS vs. DPS.

In the age ≤ 40 and > 40 years subgroups, the difference in complication incidence between the TS and DPS groups was not significant (all p > 0.05) (**Supplementary Tables 1**, **2**). In the non-radiotherapy group, there was a significant difference in the incidence of total and major complications between the TS and DPS groups but not in the radiotherapy group (all p < 0.05) (**Supplementary Tables 3**, **4**).

**Table 5** presents ORs and 95%CIs for any complications according to nipple incision suture methods with the same adjusted factors as nipple reconstruction satisfaction and the Harris scale. Compared to the SS group, the DPS group had significantly lower odds of any complications with an adjusted OR of 0.124 (95% CI: 0.018-0.863, p=0.035). Due to the small number of cases with major complications in each group, the mixed-effects logistic regression analysis of major complications was not performed.

**Table 5:** OR and 95%CI for any complications according to nipple incision suture methods.

Variables	Any complications			
	Rude OR (95%CI)	p	Adjusted OR (95%CI)	p
SS (n=17)	Reference	0.086	Reference	0.097
SPS(n=21)	1.071(0.293-3.916)	0.917	0.496(0.070-3.506)	0.482
DPS(n=50)	0.357(0.109-1.172)	0.090	0.124(0.018-0.863)	0.035

OR, Odds ratio; 95%CI, 95% confidence interval; DPS, double purse-string suture; SS, spindle suture; TS, traditional suture; BMI, body mass index; ER, estrogen receptor; PR, progesterone receptor; HER-2, human epidermal growth factor receptor-2.

Adjusted OR (95%CI): adjust for age, BMI, breast ptosis, preoperative cup size, operation time, reconstruction method, axillary surgery, extent of nipple resection, implant type, implant size, adjuvant radiotherapy and follow-up time.

**Figure 3:**
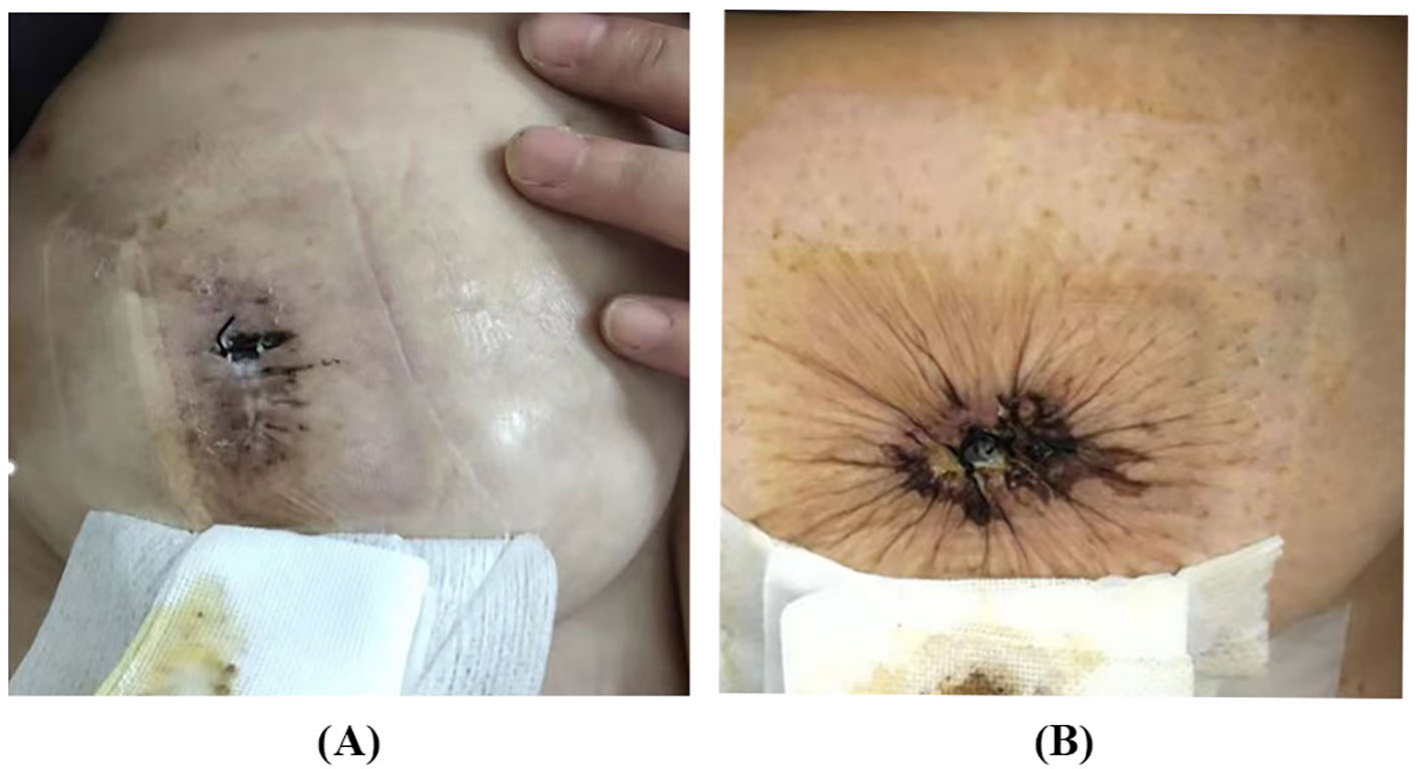
The typical images of postoperative complications. **(A)** Incision disruption after SS. **(B)** Incision disruption after SPS.

The images of typical postoperative complications are shown in **Figure 3**.

### 3.4 Oncologic safety

Our study showed that the follow-up time for the SS, SPS, and DPS groups was 33.27(28.67-35.60), 37.53(30.73-48.47) and 30.35(20.33-38.63) months (p=0.040). One patient in the SS group had vertebral metastases at 29 months after the operation, while there has been no recurrence, metastasis, or death observed in the other two groups.

## 4 Discussion

Our study aimed to investigate the safety and aesthetic outcomes of DPS in early breast cancer patients who underwent nipple resection and E-SSM with breast reconstruction. By comparing with traditional SS and SPS, we found that DPS is a constructive method for nipple incision closure and nipple reconstruction. This approach not only improves patient’s satisfaction with nipple, but also reduces the any complication incidence. Moreover, DPS does not increase patients’ oncological risk. We believe that our findings can inform clinical practice and improve outcomes for patients undergoing nipple resection.

The differences of general characteristics between the DPS and TS groups may be related to the following reasons. Firstly, regarding the follow-up time, both SPS and SS are traditional techniques, with SPS being more dated, while DPS is a novel technique applied in recent years, resulting in a significantly lower follow-up time in the DPS group. Secondly, the differences in durations of operation and hospitalization may be related to the operator’s increased proficiency in the surgical procedure as well as improvements in technique (28). From the follow-up time, it can be noticed that SPS, SS, and DPS were gradually applied to nipple incision closure. As time approached, the surgeon’s operative proficiency increases, and the operation duration gradually shortened. Meanwhile, in recent years, our hospital has conducted a series of explorations in breast endoscopic reconstructive surgery. Our team has innovatively proposed many novel techniques, such as the inverse sequence method and the small areolar incision (Huaxi hole 1), which can significantly shorten the operation duration and reduce the incidence of postoperative complications (4, 5, 27, 35). Thirdly, 24h-admission operation for endoscopic breast reconstruction is a new form developed in recent years. A few years ago, under the context of COVID-19, to shorten patient’s hospitalization duration and reduce the epidemic spread, our team attempted to apply the endoscopic breast reconstruction technique to 24h-admission operation, which has been used ever since (1). As mentioned above, DPS is also a newly applied technique, overlapping with the application of 24h-admission operation for endoscopic breast reconstruction, thus having a higher percentage of 24h-admission operation and shorter hospitalization durations. Fourthly, for positive margins of the NAC after NSM, the currently accepted approach is complete resection of the NAC. However, several previous studies have shown that nipple resection only and areola preservation have comparable oncologic outcomes (36–38). In our study, in order to reconstruct the nipple with the residual areola, the areolar was preserved as much as possible in the DPS group. Consequently, the majority of DPS patients underwent simple nipple resection from the nipple root with the assurance of negative margins. If there are wide tumor invasion in the NAC region, some patients will have a spindle resection of the nipple and all areola, even including some extra-areolar skin, which constitute the SS group. However, it is crucial to note that the extent of nipple resection affects the size of the skin defect in front of the breast, which in turn influence the incision tension and breast shape after closing. Shuai Li et al. concluded that the wound tension may be related to the size and the location, the larger the surgical margins, the greater the surgical incisional tension (39). Adequate blood supply is essential for tissue healing. Excessive incision tension may affect local blood perfusion, resulting in a lack of nutrients and oxygen, inducing inflammatory reactions and tissue necrosis (39–43). Hunt et al. reported that ischemic wounds are more susceptible to infection than well-vascularized wounds (44). Besides, when the defect is significant, the breast will be deformed, uplifted, and reduced in projection, decreasing the symmetry of the bilateral breasts. Among the three groups, DPS group had the most minor nipple resection, so that the incision tension was low. The outer purse-string suture can further close incision with uniform pulling force and reduce the tension for the inner purse-string suture, promoting the incision healing, reducing complications, and having little effect on breast shape. For the SS group, the anterior breast skin defect was so significant that the incision tension was high. The closed incision was linear and the pulling force on the front of the breast was uneven. Thus, it’s likely to have complications and breast deformation. However, the SS incision could be closed in layers to enhance the tightness of the incision closure and reduce the risk of complications. The extent of nipple resection in the SPS group was similar to that of DPS, but the incision was closed only by a single purse-string suture. The incision tension was high and the suture was underpowered, so the rate of major complications was significantly higher than in the other two groups. In SPS, as in DPS, the incision and suture were round, so the skin in front of the breast was pulled with a uniform force, and the closure had little effect on the shape of the breast. However, since the above differences in general characteristics can also affect the aesthetic outcomes and safety of the patients, we included these variables in the subsequent statistical analysis of the suture methods and the aesthetic outcomes and safety to reduce the influence on the relevant results (45–48).

Regarding aesthetic outcomes, our data show that the DPS group consistently had higher nipple reconstruction satisfaction than the SS group, with or without adjustment for covariates. About nipple reconstruction satisfaction, we think that it mainly relates to the reconstructive nipple effect that DPS has. After SS, there is no nipple or nipple-like structure, while after SPS, a round nipple-like bulge will be formed without column structure. However, the tightened DPS can improve the convexity of the nipple and areola area and shape the nipple column, resulting in nipple reconstruction, consistent with previous findings. DPS helps to reconstruct the nipple and areola and increase projection (22, 49). Kanelina Bimpa et al. reported that the DPS technique can safely and effectively close nipple incisions for nipple adenomas and reconstruct the nipple with an acceptable aesthetic result (50). The purse-string suture can help patients with inverted nipples to obtain ideal nipple shape and has the advantages of ideal nipple shape and minimal injury and scarring (20, 21).With respect to Harris scale, which is evaluated from the overall appearance of the breast symmetry, including the breasts and nipples described above (32). Concerning breast aesthetics, except for the nipple resection extent and the uniformity of tension as mentioned above, incision scar on the breast is also a major influencing factor (33). SS’s long linear scar will have a worse impact on the breast appearance compared to the punctate scar in the SPS and DPS groups. Meanwhile, the incision tension can also affect the scar. For centuries, surgeons have observed that exuberant fibrosis and pathologic scars tend to occur at sites with high tensile stress (51, 52). Based on the discussion above, SS and SPS have higher tension among the three suture methods, while DPS has the least. Our data also reflect a trend toward higher Harris scale and Scar-Q scores in DPS, which deserves further exploration. Moreover, nipple reconstruction satisfaction and Harris scale are subjective indicators of the patient’s evaluation, so it should not be ignored that complications may have an indirect negative impact on them (32, 33, 53).

Our data reflected that DPS was associated with a lower incidence of any complication, whereas SPS was related to a higher incidence of major complications. For SPS, the nipple incision was under high tension and closed with a single purse-string suture. The suture is not strong enough, thus being more likely to have complications such as incision disruption, surgical site infection, and even implant removal (39, 44). At this point, DPS adds a purse-string suture to SPS and reinforces the reconstructed nipple with several complete interrupted suture, resulting in a tighter incision closure and promoting incision healing. Thus, compared with SS, DPS has lower any complication odds, even adjusted for covariates such as nipple resection extent.

In addition, we found that age subgroups influenced the changes in complications and aesthetic outcomes associated with the nipple incision suture method. This is similar to the finding of Lai et al. that age plays a vital role in postoperative outcomes of NSM (46, 47). In the non-radiotherapy group, the DPS group had significantly better cosmetic outcomes and complication rates than the TS group, which may imply that when radiotherapy is removed as a factor, the safety and aesthetic benefits associated with the nipple incision suture method are further accentuated. This is supported by previous studies. Radiotherapy can damage local tissues and blood vessels, resulting in inadequate blood and oxygen supply and increasing the incidence of postoperative complications such as infection, incision dehiscence, NAC and flap ischemia. Radiotherapy can also cause skin pigmentation and tissue atrophy, impairing breast aesthetics (54–60). However, there is insufficient evidence in our study and these findings deserve further exploration. In this study, the potential effects of these two factors on safety and aesthetic outcomes were corrected by the mixed-effects logistic regression model.

Based on the current follow-up data, one patient in the SS group reported vertebral metastasis at 19 weeks after surgery. There were no cases of metastasis or recurrence in the DPS group. This may suggest that DPS does not increase the oncologic risk in early breast cancer patients who underwent nipple resection and E-SSM with breast reconstruction compared to other nipple suture methods.

Regarding the significant advantage of the DPS, the nipple reconstruction effect, we want to explore further. Nipple reconstruction is the finishing touch to breast reconstruction, which plays an important role in the overall aesthetic outcome of the reconstructed breast (13, 61, 62). Some researchers support that nipple reconstruction should be performed after the reconstructed breast has been adapted to the final shape and position (61, 63), while some researchers are against it. They believe that immediate nipple reconstruction effects are comparable to delayed surgery, which will bring additional trauma and financial burden to the patients and weaken the willingness of the patients to undergo nipple reconstruction (36, 63–69).

Over the past decades, many techniques for nipple reconstruction have been explored (13), such as local flap grafts, material grafts (autologous, allogeneic, synthetic), shared nipple, tattooing and so on. Compared with the complex techniques above, DPS has the following advantages. Firstly, DPS is easy to perform and can be conducted concurrently with breast reconstruction. It is an *in-situ* reconstruction using residual areolar tissue, so the position of the reconstructed nipple can be determined intraoperatively, without waiting for the reconstructed breast to be finalized. Secondly, DPS can avoid additional damage and the cost to of harvesting the graft. Thirdly, in DPS, the nipples formed by areola retraction have their color, consistent with the color of the contralateral nipples and areola and will not fade easily (36). Finally, the tumor can still be completely resected with guaranteed negative margins without increasing the risk of oncologic safety. However, every coin has two sides. The DPS technique actually has some shortcomings, such as the projection decreases over time, which is a problem common to almost all nipple reconstruction methods (13, 16, 61, 62). Our recommendation is to shape the size of the reconstructed nipple projection by controlling the spacing between the inner and outer purse strings, with a recommended distance of 1 cm. In addition, the implementation of DPS will be limited by the size of the excision area, and it is challenging to perform when the excision area is too large. A large excision area means more skin and tissue defects. Then, breast deformation is likely to occur regardless of the suture method used. In such cases, we recommend using a latissimus dorsi muscle flap for breast reconstruction, which can repair the skin defect and provide a localized flap for nipple reconstruction (28, 68, 70).

This study also has limitations, such as a small sample size and the inevitable shortcomings of a cross-sectional study. Therefore, the conclusions obtained in this study need to be further explored in a prospective cohort study or randomized controlled trial with large sample sizes and long follow-up time.

## 5 Conclusions

DPS can improve the aesthetic breast outcomes and decrease the incidence of any postoperative complications, without increasing adverse oncologic outcomes in early breast cancer patients who underwent E-SSM with breast reconstruction, which is a worthwhile surgical modality for patients with nipple resection. Besides, DPS is a promising technique for nipple reconstruction.

## Data Availability

The data used in the study are available from the corresponding author upon reasonable request.

## Supplementary material

Supplementary Table 1 Comparison of postoperative complications and aesthetic outcomes of different nipple incision suture methods in patients aged ≤40 years.

Supplementary Table 2 Comparison of postoperative complications and aesthetic outcomes of different nipple incision suture methods in patients aged >40 years.

Supplementary Table 3 Comparison of postoperative complications and aesthetic outcomes of different nipple incision suture methods in patients undergoing radiotherapy.

Supplementary Table 4 Comparison of postoperative complications and aesthetic outcomes of different nipple incision suture methods in patients without radiotherapy.
