# Supplementary material for: Safety and aesthetic outcomes of double purse-string suture nipple reconstruction in early breast cancer patients undergoing nipple resection and endoscopic skin-sparing mastectomy with breast reconstruction
Source: Front Oncol. 2024 Sep 30;14:1462850. doi: 10.3389/fonc.2024.1462850 (PMC11476627; doi:10.3389/fonc.2024.1462850)
Supplement: Supplementary Table 1 — Comparison of postoperative complications and aesthetic outcomes of different nipple incision suture methods in patients aged ≤40 years. [file Table1.docx]

#### **Supplementary Table 1.** Comparison of postoperative complications and aesthetic outcomes of different nipple incision suture methods in patients aged ≤40 years.

| Variables | TS (n=15) | | DPS (n=21) | p1 | p2 |
| --- | --- | --- | --- | --- | --- |
|  | SS (n=7) | SPS (n=8) |  |  |  |
| Any complications |  |  |  | 0.304 | 0.236 |
| Yes | 3(42.9) | 2(25.0) | 3(14.3) |  |  |
| No | 4(57.1) | 6(75.0) | 18(85.7) |  |  |
| Major complications |  |  |  | 0.417 | 0.417 |
| Yes | 0(0.0) | 1(12.5) | 0(0.0) |  |  |
| No | 7(100.0) | 7(87.5) | 21(100.0) |  |  |
| Minor complications |  |  |  | 0.229 | 0.418 |
| Yes | 3(42.9) | 1(12.5) | 3(14.3) |  |  |
| No | 4(57.1) | 7(87.5) | 18(85.7) |  |  |
| Nipple reconstruction satisfaction, n (%) |  |  |  | 0.007 | 0.002 |
| Very Satisfied | 0(0.0) | 0(0.0) | 9(42.9) |  |  |
| Relatively Satisfied | 2(28.6) | 2(25.0) | 6(28.6) |  |  |
| Relatively dissatisfied | 2(28.6) | 5(62.5) | 6(28.6) |  |  |
| Very dissatisfied | 3(42.9) | 1(12.5) | 0(0.0) |  |  |
| Harris scale, n (%) |  |  |  | 0.142 | 0.071 |
| Excellent | 1(14.3) | 0(0.0) | 9(42.9) |  |  |
| Good | 1(14.3) | 3(37.5) | 5(23.8) |  |  |
| Fair | 4(57.1) | 5(62.5) | 6(28.6) |  |  |
| Poor | 1(14.3) | 0(0.0) | 1(4.8) |  |  |
| **Abbreviations:** TS: traditional suture; SS: spindle suture; SPS: single purse-string suture; DPS: double purse-string suture  **Footnotes:** p1: SS vs. SPS vs. DPS; p2: TS vs. DPS. | | | | | |

#### **Supplementary Table 2.** Comparison of postoperative complications and aesthetic outcomes of different nipple incision suture methods in patients aged >40 years.

| Variables | TS (n=23) | | DPS (n=29) | p1 | p2 |
| --- | --- | --- | --- | --- | --- |
|  | SS (n=10) | SPS (n=13) |  |  |  |
| Any complications |  |  |  | 0.133 | 0.075 |
| Yes | 4(40.0) | 7(53.8) | 7(24.1) |  |  |
| No | 6(60.0) | 6(46.2) | 22(75.9) |  |  |
| Major complications |  |  |  | 0.093 | 0.191 |
| Yes | 0(0.0) | 2(15.4) | 0(0.0) |  |  |
| No | 10(100.0) | 11(84.6) | 29(100.0) |  |  |
| Minor complications |  |  |  | 0.317 | 0.140 |
| Yes | 4(40.0) | 6(46.2) | 7(24.1) |  |  |
| No | 6(60.0) | 7(53.8) | 22(75.9) |  |  |
| Nipple reconstruction satisfaction, n (%) |  |  |  | 0.226 | 0.184 |
| Very Satisfied | 2(20.0) | 1(7.7) | 9(31.0) |  |  |
| Relatively Satisfied | 2(20.0) | 6(46.2) | 10(34.5) |  |  |
| Relatively dissatisfied | 4(40.0) | 2(15.4) | 8(27.6) |  |  |
| Very dissatisfied | 2(20.0) | 4(30.8) | 2(6.9) |  |  |
| Harris scale, n (%) |  |  |  | 0.032 | 0.096 |
| Excellent | 5(50.0) | 2(15.4) | 15(51.7) |  |  |
| Good | 3(30.0) | 2(15.4) | 5(17.2) |  |  |
| Fair | 1(10.0) | 6(46.2) | 9(31.0) |  |  |
| Poor | 1(10.0) | 3(23.1) | 0(0.0) |  |  |
| **Abbreviations:** TS: traditional suture; SS: spindle suture; SPS: single purse-string suture; DPS: double purse-string suture  **Footnotes:** p1: SS vs. SPS vs. DPS; p2: TS vs. DPS. | | | | | |

#### **Supplementary Table 3.** Comparison of postoperative complications and aesthetic outcomes of different nipple incision suture methods in patients undergoing radiotherapy.

| Variables | TS (n=13) | | DPS (n=17) | p1 | p2 |
| --- | --- | --- | --- | --- | --- |
|  | SS (n=5) | SPS (n=8) |  |  |  |
| Any complications |  |  |  | 0.852 | 0.698 |
| Yes | 1(20.0) | 3(37.5) | 4(23.5) |  |  |
| No | 4(80.0) | 5(62.5) | 13(76.5) |  |  |
| Major complications |  |  |  | 0.433 | 0.433 |
| Yes | 0(0.0) | 1(12.5) | 0(0.0) |  |  |
| No | 5(100.0) | 7(87.5) | 17(100.0) |  |  |
| Minor complications |  |  |  | 1.000 | 1.000 |
| Yes | 1(20.0) | 2(25.0) | 4(23.5) |  |  |
| No | 4(800) | 6(75.0) | 13(76.5) |  |  |
| Nipple reconstruction satisfaction, n (%) |  |  |  | 0.384 | 0.234 |
| Very Satisfied | 0(0.0) | 0(0.0) | 3(17.6) |  |  |
| Relatively Satisfied | 2(40.0) | 6(75.0) | 6(35.3) |  |  |
| Relatively dissatisfied | 2(40.0) | 1(12.5) | 7(41.2) |  |  |
| Very dissatisfied | 1(20.0) | 1(12.5) | 1(5.9) |  |  |
| Harris scale, n (%) |  |  |  | 0.976 | 0.944 |
| Excellent | 1(20.0) | 1(12.5) | 4(23.5) |  |  |
| Good | 2(40.0) | 2(25.0) | 4(23.5) |  |  |
| Fair | 2(40.0) | 4(50.0) | 8(47.1) |  |  |
| Poor | 0(0.0) | 1(12.5) | 1(5.9) |  |  |
| **Abbreviations:** TS: traditional suture; SS: spindle suture; SPS: single purse-string suture; DPS: double purse-string suture  **Footnotes:** p1: SS vs. SPS vs. DPS; p2: TS vs. DPS. | | | | | |

#### **Supplementary Table 4.** Comparison of postoperative complications and aesthetic outcomes of different nipple incision suture methods in patients without radiotherapy.

| Variables | TS (n=25) | | DPS (n=33) | p1 | p2 |
| --- | --- | --- | --- | --- | --- |
|  | SS (n=12) | SPS (n=13) |  |  |  |
| Any complications |  |  |  | 0.043 | 0.022 |
| Yes | 6(50.0) | 6(46.2) | 6(18.2) |  |  |
| No | 6(50.0) | 7(53.8) | 27(81.8) |  |  |
| Major complications |  |  |  | 0.087 | 0.181 |
| Yes | 0(0.0) | 2(15.4) | 0(0.0) |  |  |
| No | 12(100.0) | 11(84.6) | 33(100.0) |  |  |
| Minor complications |  |  |  | 0.073 | 0.044 |
| Yes | 6(50.0) | 5(38.5) | 6(18.2) |  |  |
| No | 6(50.0) | 8(61.5) | 27(81.8) |  |  |
| Nipple reconstruction satisfaction, n (%) |  |  |  | 0.006 | 0.001 |
| Very Satisfied | 2(16.7) | 1(7.7) | 15(45.5) |  |  |
| Relatively Satisfied | 2(16.7） | 2(15.4) | 10(30.3) |  |  |
| Relatively dissatisfied | 4(33.3) | 6(46.2) | 7(21.2) |  |  |
| Very dissatisfied | 4(33.3) | 4(30.8) | 1(3.0) |  |  |
| Harris scale, n (%) |  |  |  | 0.006 | 0.008 |
| Excellent | 5(41.7) | 1(7.7) | 20(60.6) |  |  |
| Good | 2(16.7) | 3(23.1) | 6(18.2) |  |  |
| Fair | 3(25.0) | 7(53.8) | 7(21.2) |  |  |
| Poor | 2(16.7) | 2(15.4) | 0(0.0) |  |  |
| **Abbreviations:** TS: traditional suture; SS: spindle suture; SPS: single purse-string suture; DPS: double purse-string suture  **Footnotes:** p1: SS vs. SPS vs. DPS; p2: TS vs. DPS. | | | | | |
